# Rural–Urban Differences: Using Finer Geographic Classifications to Reevaluate Distance and Choice of Health Services in Malawi

**DOI:** 10.1080/23288604.2022.2051229

**Published:** 2022-01-01

**Authors:** Kaitlyn McBride, Corrina Moucheraud

**Affiliations:** Department of Health Policy and Management, UCLA Fielding School of Public Health, Los Angeles, California, USA

**Keywords:** Access to care, geographic access, healthcare choice, rural–urban classification, rural–urban differences

## Abstract

There is no universal understanding of what defines urban or rural areas nor criteria for differentiating within these. When assessing access to health services, traditional urban–rural dichotomies may mask substantial variation. We use geospatial methods to link household data from the 2015–2016 Malawi Demographic Health Survey to health facility data from the Malawi Service Provision Assessment and apply a new proposed four-category classification of geographic area (urban major metropolitan area, urban township, rural, and remote) to evaluate households’ distance to, and choice of, primary, secondary, and tertiary health care in Malawi. Applying this new four-category definition, approximately 3.8 million rural- and urban-defined individuals would be reclassified into new groups, nearly a quarter of Malawi’s 2015 population. There were substantial differences in distance to the nearest facility using this new categorization: remote households are (on average) an additional 5 km away from secondary and tertiary care services versus rural households. Health service choice differs also, particularly in urban areas, a distinction that is lost when using a simple binary classification: those living in major metropolitan households have a choice of five facilities offering comprehensive primary care services within a 10-km zone, whereas urban township households have no choice, with only one such facility within 10 km. Future research should explore how such expanded classifications can be standardized and used to strengthen public health and demographic research.

## Introduction

There is no universal understanding of what defines urban or rural areas nor criteria for differentiating within these. The United Nations recognizes that no single urban–rural definition is applicable to all countries, given national differences in characteristics that can distinguish these areas, and recommends the use of additional criteria for further classification.^1^ Therefore, specific countries apply their own criteria—based on population threshold, population density, or presence of infrastructure—and definitions vary greatly, which limit cross-national comparisons or multi-country analyses.^2–6^ This is a concern even for standardized surveys like the Demographic Health Surveys (DHS) as these adopt each country’s own urban–rural definition.

In Malawi, classified “urban” areas are the four major metropolitan areas of Lilongwe, Blantyre, Mzuzu, and Zomba, plus secondary cities, e.g., townships and district centers, while all other areas in the country are designated as rural.^7^ The four major metropolitan areas, as defined in the Malawi Population and Housing Census,^7^ host most non-agricultural economic activities (manufacturing, trading, and financial services), while Malawi’s urban townships are primarily agrarian and located at the edge of rural–urban borders.^8^ Likewise, rural Malawi—which is where approximately 84% of the population lives—is extremely varied and includes both peri-urban areas and vast regions of sparsely populated areas with limited infrastructure and road access.^9,10^

Use of a dichotomous urban–rural classification fails to capture these important variations, which may affect the availability of services and accessibility to care, and varying applications of the defining characteristics of “rural” and “urban” may lead to misclassification of urban or rural residence.^11,12^ There is a large body of research that demonstrates the critical role of geographic proximity and access to health facilities for improved care utilization and health outcomes. Access to care in urban areas is often assumed to be superior to those living in rural areas; rural residents are more likely to travel long distances for care, especially secondary and tertiary care, and have fewer options in choice of health facility.^13–16^ Compared to their urban counterparts, rural populations also experience poorer health status and outcomes, commonly referred to as the urban advantage (or rural disadvantage).^17^ However, few studies disaggregate these “urban” and “rural” groups to assess, for example, differences between major metropolitan areas and secondary cities, or across dimensions of rurality. Classifying geographic areas too coarsely into dichotomous urban–rural groups may cause misclassification errors in research and may influence program and policy decisions about resource allocation, leading to a neglect of more remote populations and therefore deepening disparities.

Understanding variation within rural and urban classifications has important implications for achieving Universal Health Coverage (UHC) and ultimately reaching global mortality reduction targets as access to essential health services can increase health service utilization and improve health outcomes. Despite recent improvements, there remain gaps in coverage of critical health services (including for HIV, TB, and malaria^18^) in many low- and middle-income countries, and these service gaps are much larger in rural and remote areas compared to urban settings.^6,19^ Expanded coverage is needed particularly in remote regions with shortages of health professionals, limited supplies, and poor transportation infrastructure.

To achieve UHC and improve gaps in care coverage, there exists a need to better understand what urban and rural means and to move away from using these measures, by expanding and refining the standard binary definition (rural versus urban).^20^ One option is to use multi-dimensional measures that incorporate both geographic and population characteristics. For example, the Index of Relative Rurality takes into account four dimensions of rurality in its multidimensional measure: population size, population density, remoteness, and built-up area, based on an index of values that range from 0 (a very low level of rurality) to 100 (a very high level), and has been applied to define degree of rurality in the US in studies evaluating health coverage and access.^20–22^ However, no such index is systematically applied in global health research.

The primary aim of this study is to reexamine the traditional binary rural–urban definition to develop a new classification that can be adopted globally, using Malawi as a case study. This is, to our knowledge, the first analysis to formally interrogate the commonly accepted and used definitions of “urban” and “rural” in health systems research in a lower-income setting. We use geospatial methods to link data census enumeration areas with the Malawi Service Provision Assessment (SPA), in order to answer the following questions: (1) What proportion of the Malawian population would be reclassified under a newly proposed categorization? (2) How well do standard rural–urban measures represent the healthcare service environment of households in Malawi? (3) Using a more granular definition, what new insights emerge about distance to, and choice of, primary, secondary, and tertiary health services?

## Materials and Methods

### Data Sources

National population data for Malawi were obtained from the WorldPop database for 2015, represented by a 100 square meter gridded population surface.^23^ The spatial locations of all enumeration area (EA) boundaries are from the 1998 Malawi Census of Population and Housing.^24^ The census sampling frame was stratified within regions and by urban–rural designation.^25^ The study sample includes 9145 EAs.

Health facility data are from the 2013–2014 Malawi Service Provision Assessment (SPA) survey, a cross-sectional census of all formal public and private sector health facilities in Malawi (including hospitals, health centers, and clinics).^26^ SPA georeferenced the exact locations of all health facilities using GPS receivers. This analysis incorporates data from all 977 health facilities included in the SPA.

Malawi road network data (primary, secondary, and tertiary roads) were obtained from OpenStreetMap datasets.^24,27^

The Global Human Footprint Index (used to evaluate health service accessibility in rural areas) is a global dataset created from nine data layers: population density, human land use and infrastructure (built-up areas, nighttime lights, and land use/land cover), and human access (coastlines, roads, railroads, and navigable rivers). Each 1-km grid cell is assigned a score from 0 (extremely rural) to 100 (extremely urban).^28^

### Data Analysis

We first classified each EA’s spatial location as rural versus urban, following Malawi’s national definition: urban areas (the four major metropolitan areas plus secondary cities, e.g., townships and district centers), and all other areas in the country were designated as rural. We then reclassified each “urban” EA as belonging to either a major metropolitan area (MMA) or secondary city/township. Among the “rural” EAs, we evaluated the degree of rurality using the Global Human Footprint Index and reclassified all EAs below the 25th percentile in the score distribution as remote. Thus, our new four-category typology of geographic locations is defined as follows: MMAs, townships, rural (non-MMA or township areas above the 25th percentile of the Global Human Footprint Index), and remote (non-MMA or township areas at or below the 25th percentile of the Global Human Footprint Index).

To estimate the number of people whose locations of residence would be reclassified using the new four-category approach, we extracted the number of people living in each 100 square meter area from the 2015 WorldPop estimates and summed the population in each old and new category of urban and rural areas.

We classified each health facility according to its level of care, per Malawi’s national guidelines:^29^ primary care (available at health posts, clinics, and health centers) and secondary (provided at community and district hospitals). Among the 977 health facilities in Malawi, 926 (95%) provided primary care services, 112 provided secondary care (11%), and 5 (0.5%) provided tertiary-level care. Each facility was assigned a score (ranging 1–100%) based on the percentage of services required to be offered by the type of facility (e.g., child vaccination services required at primary care facilities and blood transfusion services at secondary-level facilities). A health facility was considered to provide “comprehensive” services of its type (primary or secondary) if the percentage of services offered, according to SPA survey responses, was above the median among all facilities at that care level. Given the small number of tertiary-level facilities (*n* = 5), no classification was assigned, and all were included in the analysis.

From the geographic centroid of each EA (as an approximation of the average household’s location), we measured distance in kilometers to the nearest health facility using road network distance, i.e., distance based on travel paths along a network of transportation routes (highways, roads, and footpaths). This was performed using the New Closest Facility tool of Network Analyst extension from ArcGIS. Euclidean (straight-line) distance to the nearest facility was conducted if road network distance could not be calculated, e.g., unable to identify a travel route or establish exact household location; this was done for approximately 1.5% of all EAs. We calculated average, median, range, and standard deviation of distance to the closest health facility of any type and by levels. We also created a 5-km and 10-km “travel zone” around each EA and counted the number of health facilities (of any type and by levels) within these travel zones. The main analysis compared these distances to the nearest facility and the availability of facilities within these travel zones, across both old and new location classifications (urban and rural; and MMA, township, rural, and remote).

All statistical analyses were conducted in Stata (14.1) and visuals in RStudio (3.6.3). All spatial analyses and geographic mapping used ArcGIS (10.7.1).

## Results

### Reclassifying Population Areas of Residence

**Figure 1.**
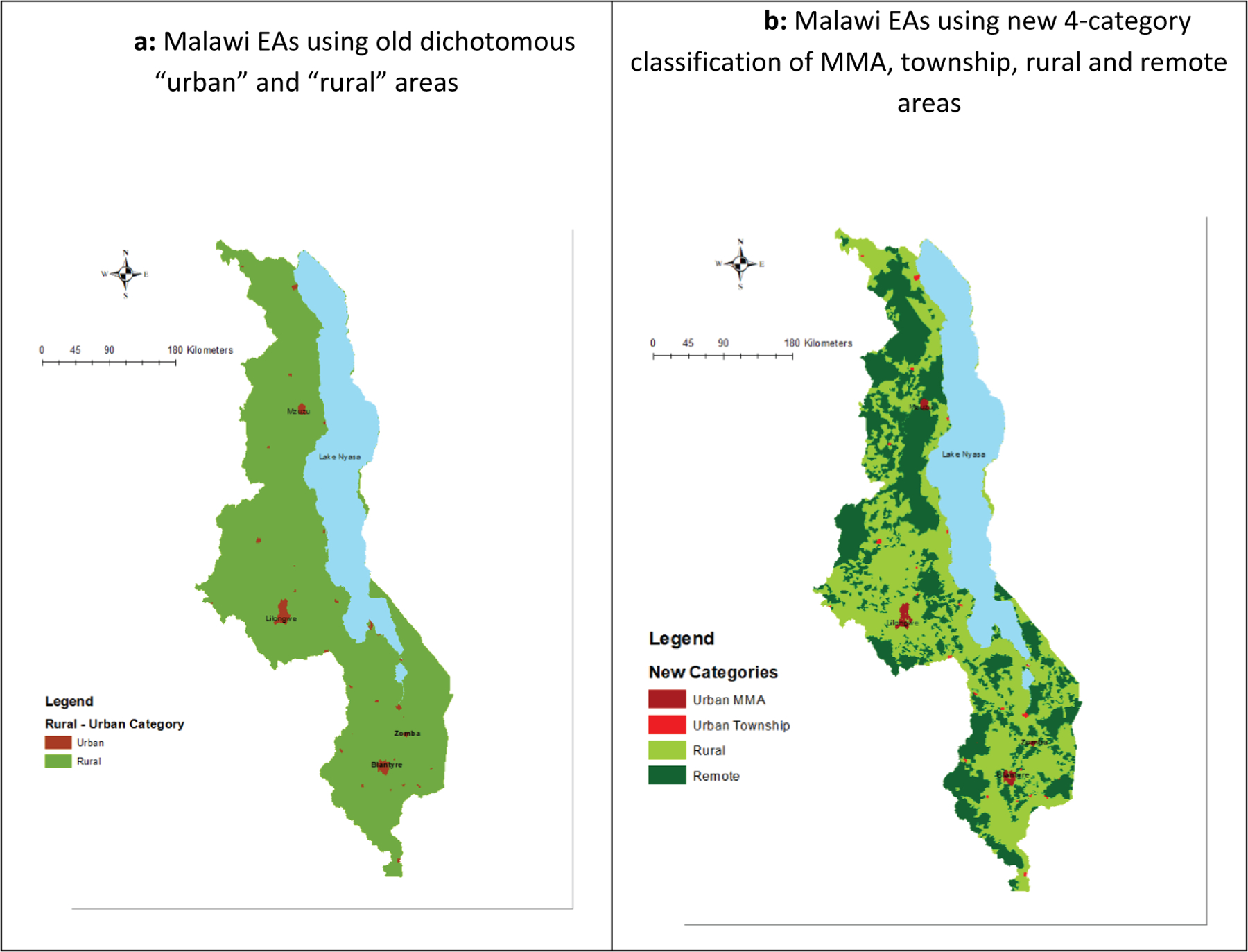
(a) Malawi EAs using old dichotomous “urban” and “rural” areas. (b) Malawi EAs using new four-category classification of MMA, township, rural, and remote areas.

Using the standard dichotomous classification, 1053 EAs were defined as “urban” and 8092 as “rural” (Figure 1a). With the new four-category classification, there were 802 MMA areas, 251 township areas, 6017 rural areas, and 2075 remote enumeration areas (Figure 1b).

In 2015, using the dichotomous definition, an estimated 84.5% of Malawians lived in “rural” EAs. By applying the new four-category typology, 25.2% of these “rural” residents—or 3.3 million people—would be reclassified as living in remote areas. Of the approximately 2.4 million Malawians living in “urban” areas, most (79.7%) were in the MMAs (Lilongwe, Blantyre, Mzuzu, and Zomba); and the remaining 480,900 people would be reclassified as living in townships based on the four-category approach.

### Distance to Health Facilities

Overall, 516 primary health facilities and 61 secondary-level facilities were identified as providing comprehensive services. The median percentage of required services provided was 86% among primary care facilities and 91% among secondary care facilities; however, the percentage of services offered across facilities varied substantially, ranging from 13% to 100%.

**Figure 2.**
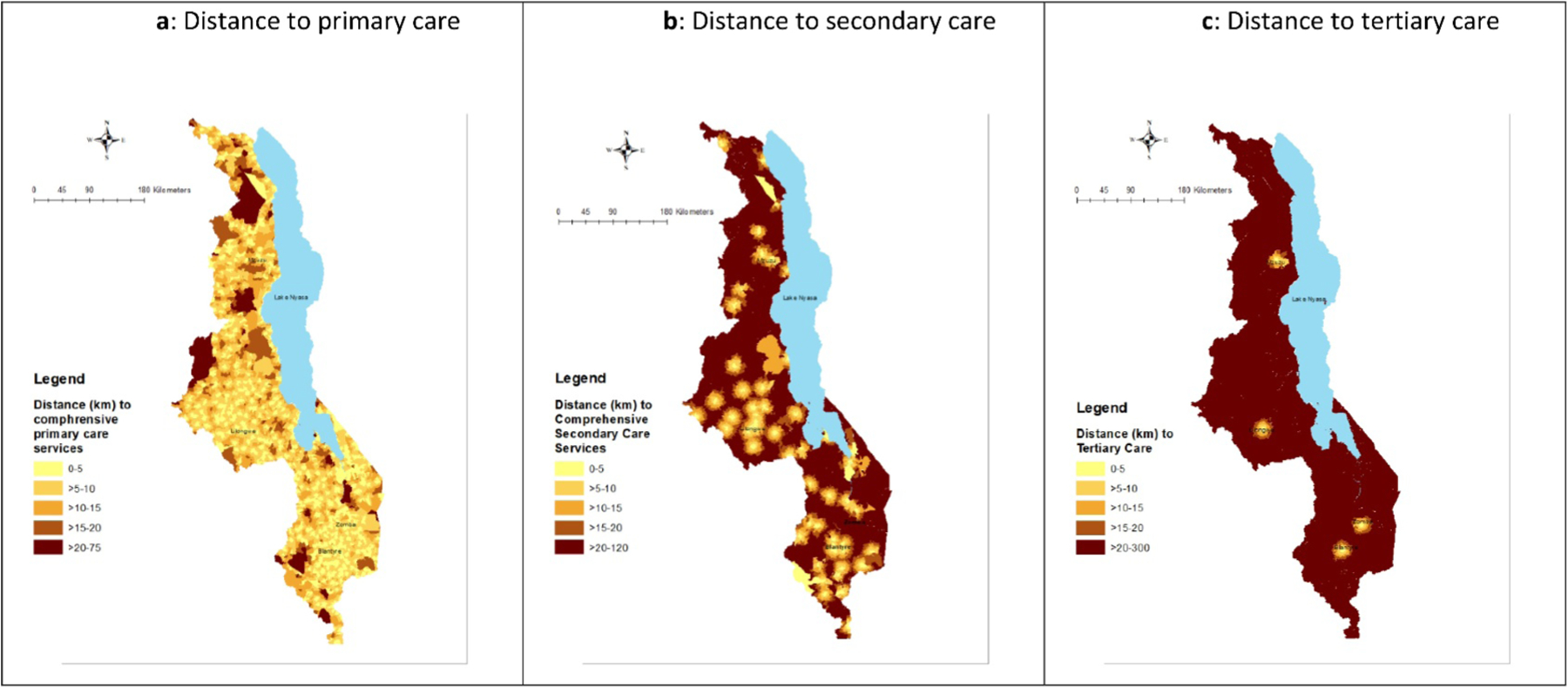
(a) Distance to primary care. (b) Distance to secondary care. (c) Distance to tertiary care.

Most households in Malawi live within 5 km of a health facility offering comprehensive primary care services (Figure 2a). The range in distance to care is greatest among remote households (<1 to 32 km) (Appendix Table A1). Access to facilities offering comprehensive secondary and tertiary care diminishes across all households, particularly among those outside cities (Figure 2b,c). Only MMA households are within 15 km of tertiary care.

**Figure 3.**
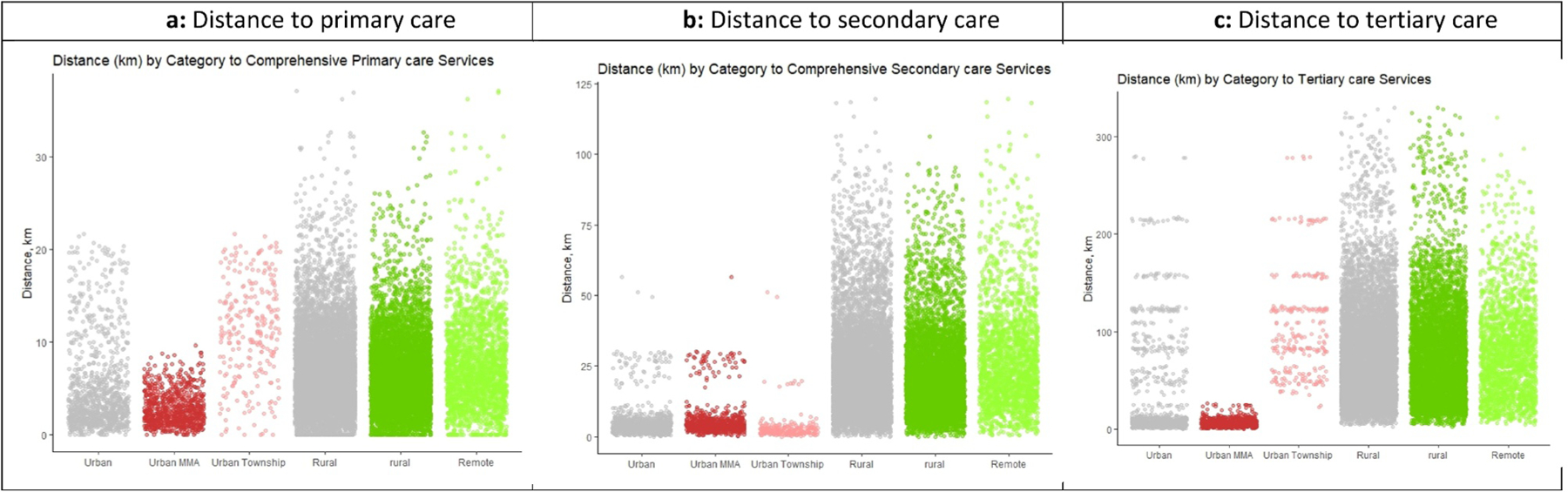
(a) Distance to primary care. (b) Distance to secondary care. (c) Distance to tertiary care.

The average distance to comprehensive primary-level services varied the greatest between urban MMA and township households, at 2.8 and 11.2 km, respectively (Figure 3a; Appendix Table A1). Among MMA households, the greatest distance to care was 9.6 km and 21.6 km among township households. Using the previous dichotomous “urban” definition, the average distance is 4.8 km.

The average distance to comprehensive secondary-level services for a remote household is 28 km, which is considerably longer than the average distance for households classified as “Rural” using the dichotomous definition (approximately 22.8 km) (Figure 3b; Appendix Table A2). Geographic disparities in distance to tertiary care also widen with the new classification, with an additional 4.1 km to the nearest facility offering comprehensive tertiary care comparing remote to rural and an additional 99.1 km comparing MMAs to townships (Figure 3c; Appendix Table A3).

### Health Service Choice

Households outside MMAs—including urban townships (and rural and remote areas)—have at most one health facility providing comprehensive primary care services within a 5-km radius, while households within MMAs have choices (Table 1).

When we extended the radius to a 10-km zone, households in MMAs can access even more facilities offering comprehensive primary care (Table 1), but other households still only had at most one facility of any type nearby. This gap in health service choice widens when evaluating choice of secondary and tertiary care services: rural and remote households have no choice (on average) of facilities providing comprehensive secondary care services within a 10-km distance (Table 2), and only households located in MMAs have any facility providing tertiary care within 10 km (Table 3).

## Discussion

These results illustrate that traditional urban–rural classifications of households are overly coarse and miss important heterogeneity when considering access to health services. Consistent with prior studies, we find that health facilities, particularly hospitals that provide higher-level care, are concentrated in Malawi’s four MMAs.^9,30^ However, by disaggregating “urban” residents into two groups, we gain new insights about the many people—nearly half a million Malawians in secondary cities—who are similar to their MMA-dwelling counterparts in only needing to travel a short distance to access primary and secondary care but differ in having no choice of where to go. Only households in the MMAs have any choice when accessing primary and secondary care services. The standard “urban” classification does not allow this potentially very important distinction to emerge.

In addition, when we applied our new four-category typology to the 2015 national population estimate data, more than 3 million Malawians were reclassified as residing in remote areas. This underscores the importance of taking a more nuanced view to rurality as well—particularly for understanding distance to care. Studies have shown that even small distances of even just 1 or 2 km can affect care-seeking behavior and ultimately health outcomes.^13,14,16,31^ In many countries, including Malawi, rural populations face substantial transportation barriers.^32^ Poor road conditions, terrain, and lack of road infrastructure can be a significant challenge, and some roads may only be accessible by foot or bicycle; private transport is also expensive, and the majority of rural populations lack access to a vehicle.^9,17^

The study findings highlight the importance of shifting away from traditional urban–rural classification to evaluate disparities in care utilization and outcomes (geospatial methods that incorporate precise location information would offer even more granularity still). A large number of existing studies (using DHS data or similar nationally representative surveys) have applied traditional rural–urban criteria to examine disparities in health outcomes;^33–40^ health care utilization and coverage (e.g., childhood immunization and maternal health services);^41–46^ and the quality of health services.^47^ However, the use of a binary rural–urban measure mispresents populations with different access to care. For example, rural populations that live on the border of a major city or township are in close proximity to care versus rural residents living far away from main centers and secondary cities. Thus, these studies may not capture the extent of the “rural disadvantage” and the true urban–rural gap.

A number of studies have shown that compared to their urban counterparts, rural and remote populations experience worse access to quality care and have poorer health outcomes.^17,39,48^ However, few studies have explored disparities in care among just rural households,^49^ where substantial access inequities exist: households in remote areas may face even greater challenges to obtaining quality services and care from skilled healthcare workers than rural populations living closer to urban centers.

Additionally, few studies have investigated whether inequities exist within traditionally urban-defined locations.^50^ Our findings highlight choice disparities between households located in urban metropolitan areas versus those located in townships, which are located substantially farther from facilities that provide specialized health services—a finding that may be overlooked in studies applying the traditional urban categorization. MMA residents who have a choice of several providers may be able to make more nuanced decisions about care-seeking based on, for example, the reputation and quality of health providers.^51^

Research disaggregating geographic and health inequalities between rural and urban populations can also guide efforts to ensure equitable distribution of healthcare services.^52^ For example, a recent study in Malawi evaluating malaria risk found that the application of traditionally defined rural–urban categories is unable to identify the social and economic factors driving risk—and suggested that classification of malaria risk along an “urban-to-rural” continuum could help policy makers and government officials to better target investment and lead to more cost-effective approaches for reducing malaria risk.^53^ In addition, by further disaggregating rural–urban classifications, we can gain important insights to inform decisions about health facility placement or alternative approaches for expanding care coverage. In Malawi, health-surveillance assistants (HSAs) play a critical role in providing primary care services, particularly in rural and remote regions.^54^ Applying a more nuanced definition of rurality can better identify remote populations with low facility coverage; HSA expansion in these areas may prove to be the most cost-effective approach to improve access and quality of primary health care, rather than placement of a new facility.

Although this study only reexamined the implications of the traditional binary rural–urban classification in Malawi, our findings should inform research in other settings about health service coverage and use. Given the rise of electronic data collection using handheld devices, more household surveys are collecting precise location data, which is more informative for understanding geospatial relationships and conducting analyses than broad groupings whether dichotomous or even our four-category typology. However, many analyses will continue to use location categories (e.g., for summary statistics), and these results suggest that even a slightly more nuanced four-category approach—major metropolitan areas, townships, rural, and remote—can deepen our understanding of access to and choice of health services.

Some limitations to this analysis should be noted. First, we did not test other characterizations of “remote” households (e.g., metrics beyond the Global Human Footprint Index). Other multi-dimensional indices that incorporate infrastructure and population density, such as the Global Human Settlement Layer, could be applied to investigate heterogeneity within rural areas. Future studies should compare different indices, especially with multi-country datasets, as some indices may be more applicable across countries or particular settings. Second, we did not have access to enumeration boundaries from the most recent 2018 Malawi census, which would provide more precise estimates of current household location and population accessibility to health services. Third, we did not evaluate the quality of services provided or whether facilities have the requisite resources or infrastructure to offer specified care functions. Quality of care is likely an important determinant of utilization (whether and where people seek care).^55^ Additionally, our analysis focused on access to comprehensive care, rather than “any care,” which may impact study findings for average distance to facilities by place of residence, as well as choice of facilities, by care level. We also did not measure utilization, which would provide additional insight into how geographic classifications represent disparities in care use. Future analyses should continue to examine the relationships between distance, care quality, and care use in the context of geographic heterogeneity—and further expand upon our findings by evaluating health outcomes between dichotomous and expanded residence classifications. Lastly, our estimates of travel “zones” did not account for seasonal effects on road conditions or differences in mode of transport, so may be overly coarse approximations of a household’s accessible facilities.

## Conclusions

Distance to health services is known to be a key barrier to accessing care. In this application of a new four-category typology of geographic location, we find that this additional nuance sheds new light on distance to services and facility choice for households traditionally classified as “rural” and “urban,” respectively. By further disaggregating data in this way, we might better identify populations with greater need and better estimate the magnitude of geographic inequities. Such classifications also have important implications for healthcare planning and resource allocation, and we encourage further study and exploration of how definitions can be improved, standardized, and used more consistently in the field of public health and demographic research.

## Supplementary Material

Supplementary Material

## Figures and Tables

**Table 1.** Number of facilities providing comprehensive primary care services for 5- and 10-km service area.

Category	*N*	Mean	(sd)	Min	Max	Median
5-km Service area						
Remote	2075	.518	.583	0	3	0
Rural	6017	.605	.596	0	4	1
Urban MMA	802	1.878	1.016	0	5	2
Urban township	251	.191	.451	0	3	0

10-km Service area						
Remote	2075	1.456	1.053	0	6	1
Rural	6017	1.626	1.008	0	7	1
Urban MMA	802	5.271	2.157	1	10	5
Urban township	2075	1.456	1.053	0	6	1

**Table 2.** Number of facilities providing comprehensive secondary care services for 5- and 10-km service area.

Category	*N*	Mean	(sd)	Min	Max	Median
5-km Service area						
Remote	2075	.057	.233	0	1	0
Rural	6017	.083	.282	0	2	0
Urban MMA	802	1.112	.736	0	4	1
Urban township	251	1.024	.379	0	2	1

10-km Service area						
Remote	2075	.19	.415	0	2	0
Rural	6017	.302	.536	0	3	0
Urban MMA	802	2.753	1.467	0	6	3
Urban township	251	1.072	.362	0	2	1

**Table 3.** Number of facilities providing tertiary services for 5- and 10-km service area.

Category	*N*	Mean	(sd)	Min	Max	Median
5-km Service area						
Remote	2075	.001	.031	0	1	0
Rural	6017	.001	.026	0	1	0
Urban MMA	802	.363	.481	0	1	0
Urban township	251	0	0	0	0	0

10-km Service area						
Remote	2075	.009	.093	0	1	0
Rural	6017	.012	.109	0	1	0
Urban MMA	802	.813	.39	0	1	1
Urban township	251	0	0	0	0	0
