# Supplementary material for: Rural–Urban Differences: Using Finer Geographic Classifications to Reevaluate Distance and Choice of Health Services in Malawi
Source: Health Syst Reform. Author manuscript; Available in PMC 2023 Mar 8. (PMC9995164; doi:10.1080/23288604.2022.2051229)
Supplement: Supplementary Material [file NIHMS1870766-supplement-Supplementary_Material.docx]

**Table A1:** Summary statistics of distance (km) to nearest facility (km) for comprehensive primary care services

| Category | N | mean | sd | Median | min | max |
| --- | --- | --- | --- | --- | --- | --- |
| Rural | 8217 | 6.932 | 4.489 | 6.191 | 0 | 37.09 |
| rural | 6089 | 6.62 | 4.174 | 6.035 | 0 | 32.612 |
| Remote | 2128 | 7.823 | 5.187 | 6.795 | 0 | 37.09 |
| Urban | 1065 | 4.831 | 4.729 | 3.057 | 0 | 21.653 |
| Urban MMA | 810 | 2.81 | 1.821 | 2.359 | 0 | 9.663 |
| Urban Township | 255 | 11.248 | 5.361 | 11.23 | 0 | 21.653 |

**Table A2:** Summary statistics of distance (km) to nearest facility (km) for comprehensive secondary care services

| Category | N | mean | sd | Median | min | max |
| --- | --- | --- | --- | --- | --- | --- |
| Rural | 8092 | 23.813 | 16.117 | 20.143 | .053 | 119.445 |
| rural | 6017 | 22.345 | 14.97 | 19.093 | .053 | 106.267 |
| Remote | 2075 | 28.072 | 18.407 | 23.931 | .528 | 119.445 |
| Urban | 1053 | 5.106 | 6.021 | 3.44 | .014 | 56.423 |
| Urban MMA | 802 | 5.69 | 6.114 | 3.907 | .303 | 56.423 |
| Urban Township | 251 | 3.238 | 5.309 | 2.129 | .014 | 51.083 |

**Table A3:** Summary statistics of distance (km) to nearest facility (km) for tertiary care

| Category | N | mean | sd | Median | min | max |
| --- | --- | --- | --- | --- | --- | --- |
| Rural | 8092 | 79.467 | 51.402 | 69.568 | 2.992 | 329.355 |
| rural | 6017 | 78.424 | 51.876 | 67.175 | 2.992 | 329.355 |
| Remote | 2075 | 82.49 | 49.891 | 75.966 | 4.652 | 319.797 |
| Urban | 1053 | 30.723 | 49.993 | 8.195 | .48 | 279.627 |
| Urban MMA | 802 | 7.094 | 4.22 | 6.135 | .48 | 26.836 |
| Urban Township | 251 | 106.224 | 54.273 | 92.773 | 23.278 | 279.627 |
